# Supplementary material for: Phylogenetic Groups, Pathotypes and Antimicrobial Resistance of Escherichia coli Isolated from Western Lowland Gorilla Faeces (Gorilla gorilla gorilla) of Moukalaba-Doudou National Park (MDNP)
Source: Pathogens. 2022 Sep 23;11(10):1082. doi: 10.3390/pathogens11101082 (PMC9607589; doi:10.3390/pathogens11101082)
Supplement: Supplementary file 1 [file pathogens-11-01082-s001.zip › pathogens-1822342-supplementary.pdf]

Table S1. Target genes and primers used for the detection of *E. coli* phylogenetic groups [52,53] and pathotypes [124].

**Primers**	**Sequences**		**Product size**	**Reference**
Phylogroups				
ChuA.1	5'-GACGAACCAACGGTCAGGAT-3'		279-bp	[52]
ChuA.2	5'-TGCCGCCAGTACCAAAGACA-3'		279-bp	[52]
YjaA.1	5'-TGAAGTGTCAGGAGACGCTG-3'		211-bp	[52]
YjaA.2	5'-ATGGAGAATGCGTTCCTCAAC-3'		211-bp	[52]
TspE4C2.1	5'-GAGTAATGTCGGGGCATTCA-3'		152-bp	[52]
TspE4C2.2	5'-CGCGCCAACAAAGTATTACG-3'		152-bp	[52]
Pathotypes				
PCR Set-1				
pcvd432	CTGGCGAAAGACTGTATCAT	EAEC	194 bp	[125]
	AAATGTATAGAAATCCGCTGTT			
lt	ACGGCGTTACTATCCTCTC	ETEC	273 bp	[124]
	TGGTCTCGGTCAGATATGTG			
stp	TCTTTCCCCTCTTTTAGTCAG	ETEC	166 bp	[124]
	ACAGGCAGGATTACAACAAAG			
sth	TTCACCTTTCCCTCAGGATG	ETEC	120 bp	[126]
	CTATTCATGCTTTCAGGACCA			
eae	TCAATGCAGTTCCGTTATCAGTT	EPEC/EHEC	482 bp	[125]
	GTAAAGTCCGTTACCCCAACCTG			
bfp	GGAAGTCAAATTCATGGGGGTAT	Typical EPEC	300 bp	[127]
	GGAATCAGACGCAGACTGGTAGT			

Legend: DECs: Diarrheagenic *E. coli*; EAEC: Enteroaggregative *E. coli;* EHEC: Enterohemorrhagic *E. coli;* EIEC: Enteroinvasive *E. coli*; EPEC: Enteropathogenic E. coli; ETEC: Enterotoxigenic E. coli; tEPEC: Typical enteropathogenic *E. coli*.
